# The KLK5 protease suppresses breast cancer by repressing the mevalonate pathway

**DOI:** 10.18632/oncotarget.1235

**Published:** 2013-09-03

**Authors:** Georgios Pampalakis, Osahon Obasuyi, Olga Papadodima, Aristotelis Chatziioannou, Vassileios Zoumpourlis, Georgia Sotiropoulou

**Affiliations:** ^1^ Department of Pharmacy, University of Patras, Rion-Patras 26500; ^2^ National Hellenic Research Foundation, Athens 11635, Greece

**Keywords:** Kallikrein-related peptidase 5 (KLK5), breast cancer, mevalonate pathway, oncogenic signaling

## Abstract

Kallikrein-related peptidase 5 (KLK5) displays aberrant expression in cancer. However, any functional association is missing. Here, we show that reconstitution of KLK5 expression in non-expressing MDA-MB-231 breast cancer cells suppresses malignancy *in vitro* and *in vivo* dose-dependently. Reactivation of KLK5 suppressed key EMT genes. Unexpectedly, we identified altered expression of genes encoding enzymes of the mevalonate pathway typical of those observed upon cholesterol starvation. Consistently, we found that *SREBF1*, the master regulator of the mevalonate pathway was induced. KLK5 re-expression leads to reduced cellular cholesterol and fatty acid synthesis and enhanced uptake of LDL-cholesterol. Suppression of the mevalonate pathway in KLK5 transfectants was further shown by reduced synthesis of isoprenoids. Indeed, we found diminished levels of active RhoA, a signaling oncoprotein that requires prenylation for activation. We propose that reduced RhoA activation plays a dominant role in suppression of malignancy by KLK5, since geranylgeranyl pyrophosphate restored active RhoA in KLK5-reverted cells resulting in increased malignancy. For the first time, we suggest that a protease may suppress breast cancer by modulating the mevalonate pathway.

## INTRODUCTION

Human kallikrein-related peptidase 5 (KLK5), which was originally identified as the stratum corneum tryptic enzyme (SCTE) [1], is an active serine protease [1-2]. Reportedly, KLK5 displays reduced or inactivated expression in breast cancers [3-5] but the potential functional consequences in tumor development and/or progression are still unknown/yet to be described. Here, we show that KLK5 may act to suppress breast cancer by inhibiting EMTs and, surprisingly, by repressing the mevalonate pathway of cholesterol metabolism. Altered metabolism in cancer cells has been known for many years but the intricacies of how metabolic pathways interconnect with oncogenic signaling remain largely untackled. The mevalonate pathway of cholesterol biosynthesis represents a central and well-described metabolic route that uses mevalonate for synthesis of isoprenoids, precursors of cholesterol, ubiquinone etc, which are also needed for post-translational prenylation of proteins. The rate-limiting step of the mevalonate pathway is the reduction of 3-hydroxy-3-methyl-glutaryl-CoA which is catalyzed by the enzyme HMGCR [6], notably, the pharmacological target of statins, the widely prescribed cholesterol-lowering drugs. Deregulation of the mevalonate pathway, achieved by ectopic expression of either full-length HMGCR or its more recently described splice variant, is causally linked to malignant transformation of the mammary gland, which pinpointed HMGCR as a candidate metabolic oncogene [7]. It is well-established that cancer cells rely on isoprenylated molecules for their growth, since prenylation (farnesylation, geranylgeranylation) of specific proteins is required for activation of oncogenic signaling [8]. On the other hand, it is now recognized that proteases are not just degrading enzymes but they play complex and quite versatile roles in cancer growth and progression, and it has been shown that specific proteases can function as tumor-suppressors [9]. As signaling molecules proteases crosstalk with kinases in complex regulatory networks [10]. *In vitro* studies indicated that KLK5 can activate the protease-activated receptors (PARs) [11-12]. For the first time and quite initriguingly our findings link KLK5, an extracellular protease, to cancer cell metabolism and indicate that inhibition of the mevalonate pathway may represent a novel mode of tumor suppression by proteases and a potential pharmacological target for anticancer therapy.

## RESULTS

### KLK5 is inactivated in breast cancer

**Figure 1:**
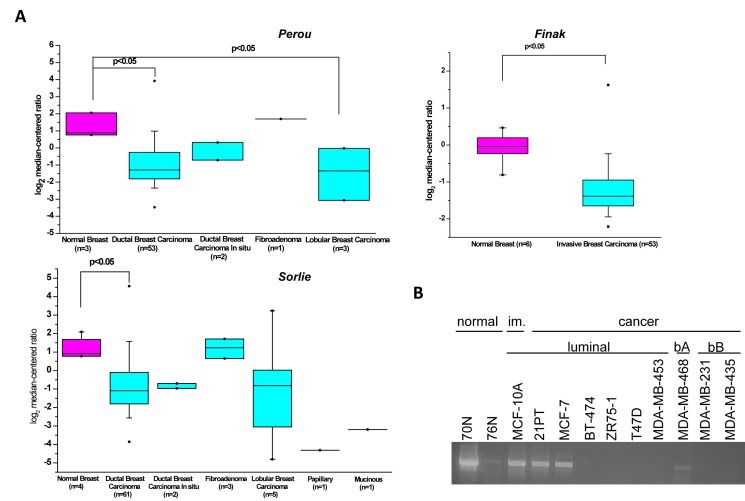
Expression of KLK5 is inactivated in breast cancer A, Box plot of KLK5 expression between normal and breast cancer cells generated by Oncomine analysis of microarray data [13-15]. B, RT-PCR analysis of KLK5 expression in normal breast cell strains, immortalized (im.) and breast cancer cell lines. bA basal A, bB basal B.

Oncomine analysis of data derived from microarray-based gene expression profiling studies employing established patient datasets [13-15] showed that *KLK5* expression is significantly down-regulated or completely inactivated in the majority of breast cancers of different subtypes compared to normal tissues (Fig.1A). This was in agreement with diminished or completely inactivated expression of *KLK5* in a panel of normal breast cell strains and tumor cell lines (Fig.1B). These findings and earlier observations [3-5, 16] suggested potential functional role(s) of KLK5 in breast cancer growth.

### Restoration of KLK5 expression reverses the malignant phenotype of MDA-MB-231 cells

**Figure 2:**
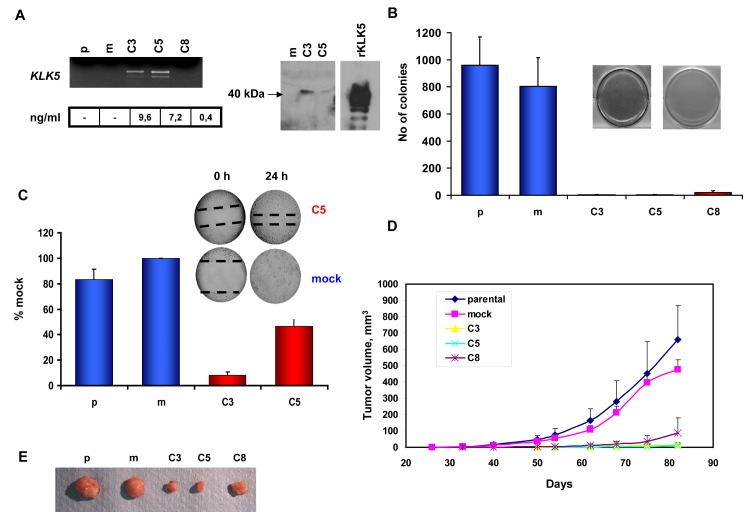
Re-expression of KLK5 in MDA-MB-231 cells suppresses their malignant phenotype A, Expression of KLK5 mRNA and protein was restored in non-expressing MDA-MB-231 cells by cDNA transfection. Three stably transfected clones were established: the C3 and C5 clones that express KLK5 at normal/physiological levels and the C8 clone that expresses low (i.e. sub-physiological) levels of KLK5. KLK5 mRNA was quantified by semi-quantitative RT-PCR; protein concentrations were measured by a specific and sensitive ELISA. The depicted Western Blot shows that the endogenous KLK5 protein secreted by C3 and C5 cells had an apparent MW of ~40 kDa, while recombinant KLK5 (rKLK5) produced in Pichia pastoris yielded four bands due to differential glycosylation. B, Restoration of KLK5 remarkably inhibited anchorage-independent growth of MDA-MB-231 in soft agar assays (bars indicate standard deviation of 5-6 replicate experiments). C, KLK5 reduces the motility of MDA-MB-231 in wound healing assays. D, Growth rates of tumor xenografts in SCID mice. KLK5 significantly represses growth of KLK5-transfectants (C3, C5, and C8) in vivo as compared to MDA-MB-231 parental and mock controls (bars indicate standard deviation of tumor volumes, n=16 sites injected). E, Representative images of tumors excised after the experimental endpoint. p: parental, m: mock.

We aimed to study the putative effects of restored KLK5 expression on the phenotype of the highly aggressive MDA-MB-231 breast cancer cell line in which the expression of KLK5 is completely inactivated. Three clones (C3, C5, and C8) transfected with the *KLK5* cDNA to stably express KLK5 mRNA and protein (Fig.2A) were established, propagated and characterized. KLK5 appeared as a single band (Fig.2A) with apparent molecular weight of ~40 kDa instead of inferred 25 kDa indicating that the protein is heavily glycosylated as also observed before [17]. In contrast, recombinant KLK5 protein produced in *Pichia pastoris* appeared as four distinct bands due to differential glycosylation at the four predicted glycosylation sites [2]. No significant differences were observed in *in vitro* proliferation rates or saturation density between parental, mock and KLK5-expressing (C3, C5, and C8) clones as they all displayed similar population doubling times (data not shown) and grew to a 100% confluence. However, KLK5 markedly inhibited anchorage-independent growth, as the C3, C5, and C8 clones failed to grow on soft agar (Fig.2B). In addition, they exhibited reduced motility in wound healing assays (Fig.2C) and significantly delayed onset of tumor formation and slower growth rates (Fig.2D; Sup Table S1) when xenotransplanted in SCID mice. Tumor volumes at the experimental endpoint (82 days post-implantation) were more than 35-fold smaller for clones C3 and C5 expressing normal concentrations of KLK5 protein compared to parental and mock (Fig.2D and E; Sup Table S1). Notably, clone C8 that produces sub-physiological concentration of KLK5 was suppressed to a lesser extend in terms of tumor onset and final sizes by approximately 6-fold smaller indicating that the tumor-suppressing effects of KLK5 are dependent on its concentration.

### Differential expression profiling-The “KLK5 signature”

**Figure 3:**
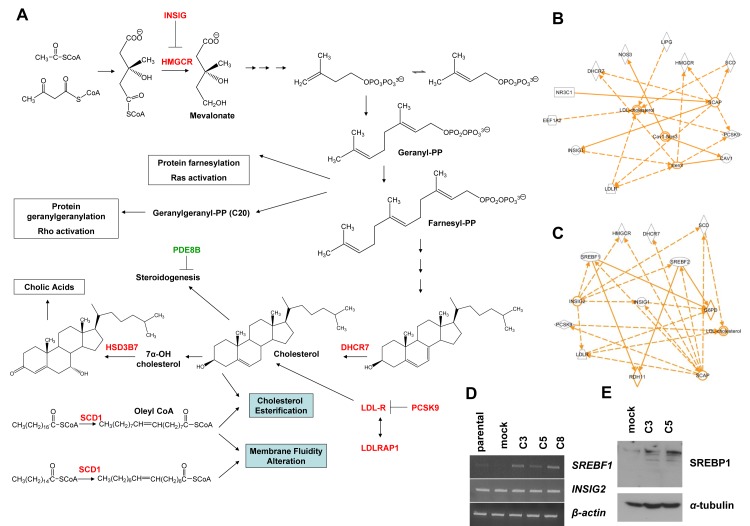
KLK5-expressing cells display altered expression of mevalonate pathway genes A, Schematic representation of the mevalonate pathway of cholesterol biosynthesis and biotransformation. Genes that are upregulated in response to KLK5 are denoted in red and downregulated genes in green. To generate an interaction network with IPA, a subset of the identified genes was selected based on their predicted functions in cholesterol and lipid metabolism (B) or genes predicted to be regulated by SREBF1 and SREBF2 (C). D, SREBF1 is activated in KLK5-expressing cells as confirmed here by RT-PCR, while the expression of INSIG2 was unaltered. E, SREBP1 is induced in KLK5-expressing cells as confirmed here by Western blotting.

With the aim to identify molecular alterations induced by KLK5 that may underlie its suppressive effects on the aggressive phenotype of MDA-MB-231 cells, we compared the gene expression profiles of MDA-MB-231 parental and mock with the profile of pooled C3 and C5 clones. The complete set of genes altered upon re-expression of KLK5 (*i.e.* the “KLK5-responsive genes” or “KLK5 signature”) consisted of 491 genes; of these, 486 genes (535 Illumina probes) were identified by microarray profiling (Sup Table S2) and 5 genes (*SNAIL1, VIM, MMP-3, LDLRAP1, SREBF1*) were tested by RT-PCR (Sup Table S3). Differential expression of selected candidate genes was confirmed by semi-quantitative RT-PCR using gene-specific primers (Sup Fig. S1). Interestingly, the “KLK5 signature” (Table 1, Sup Table S4) comprised several genes encoding key enzymes of the mevalonate pathway that leads to the biosynthesis of cholesterol and isoprenoids (Fig.3A). The list of differentially expressed genes was subjected to statistical enrichment analysis, using StRAnGER (http://www.grissom.gr/stranger/), to highlight significantly altered cellular processes by Gene Ontology (GO). Results are shown in Sup Table S4. The microarray gene list was also introduced in Ingenuity Pathway Analysis (IPA) for network construction. The obtained top network is functionally associated with lipid metabolism (Sup Fig.S2), while several gene subsets were found that control the levels of cellular cholesterol (Fig.3B and C), steroidogenesis, and the levels of phosphatidic acid. IPA prediction of transcription factor activation/inhibition against our microarray list identified activation of *SREBF1* (z=2.571) and *SREBF2* (z=2.358) transcription factors both of which interacted with other identified key genes (Fig.3B and C) as for example the *HMGCR*, *DHCR7*, *INSIG1*, etc. Consistent with the fact that transcriptional activation of *SREBF1* proceeds *via* an autoregulatory loop [18] we found markedly increased *SREBF1* mRNA and SREBP1 protein in KLK5-expressing cells (Fig.3D and E). Notably, *SREBF1* was reported by Hirsch et al. [19] to link cancer with lipid metabolism, cholesterol biosynthesis and atherosclerosis. By RT-PCR we tested the expression of *INSIG2* encoding a protein homologous to *INSIG1* that also regulates SREBPs [20] but its expression is not depended on SREBPs. Consistently, *INSIG2* was not altered (Fig.3D).

**Table 1:** Biochemical pathways and functions affected by KLK5 A total of 486-genes responsive to KLK5 were determined by oligonucleotide microarray profiling (Illumina platform). Shown here are selected up- and down-regulated genes and their corresponding functional annotation. The complete list of differentially expressed genes is given in Sup Table S2

Gene	Name	Fold change	
		UP	DOWN
Angiogenesis			
THBS2	Thrombospondin 2	16,05	
Cholesterol and Lipid Metabolism			
PCSK9	Proprotein convertase subtilisin/kexin type 9	8,69	
INSIG1	Insulin induced gene 1	7,20*	
SCD	Stearoyl-CoA desaturase (delta-9-desaturase)	4,99	
LIPG	Endothelial lipase	2,68*	
DHCR7	7-dehydrocholesterol reductase	2,33*	
LPIN1	Lipin 1	2,12	
HMGCR	3-hydroxy-3-methylglutaryl-coenzyme A reductase	2,05	
LDLR	Low density lipoprotein receptor	1,97	
HSD3B7	Hydroxy-delta-5-steroid dehydrogenase, 3 beta- and steroid delta-isomerase 7	1,77	
VAMP4	Vesicle-associated membrane protein 4	1,64	
PDE8B	Phosphodiesterase 8B		4,35
ACOT9	Acyl-CoA thioesterase 9		1,89
PLCG1	Phospholipase C, gamma 1		1,64
Inflammation			
CX3CL1	Chemokine (C-X3-C motif) ligand 1		3,85
NOS3	Nitric oxide synthase 3		3,57
Proteolysis and Protease Inhibitors			
MMP-9	Matrix metallopeptidase 9		3,03
CTSL2	Cathepsin L2		1,92
TIMP1	TIMP metallopeptidase inhibitor 1		1,85
PLAUR	Plasminogen activator, urokinase receptor		1,59
DNA Repair and Recombination			
RAD51C	RAD51 homolog C (S. cerevisiae)		3,85*
HMGB1	High-mobility group box 1	1,77	
Positive Regulation of I-kappaB Kinase/NF-kappaB Cascade			
TRIM13	Tripartite motif-containing 13	1,93*	
GPR177	G protein-coupled receptor 177	1,67*	
Nuclear proteins			
TSPYL5	TSPY-like 5 (TSPYL5)		7,14
EEF1A2	Eukaryotic translation elongation factor 1 alpha 2		4,55
CITED4	Cbp/p300-interacting transactivator, with Glu/Asp-rich carboxy-terminal domain, 4		3,03
SH3KBP1	SH3-domain kinase binding protein 1		2,22*
ELK1	Member of ETS oncogene family		1,85
CCNA1	Cyclin A1	2,34	
ELF1	E74-like factor 1 (ets domain transcription factor)	2,17	
DNMT3B	DNA (cytosine-5-)-methyltransferase 3 beta	1,59	
PDCD4	Programmed cell death 4	1,64	

* Mean fold-change (up or down) for genes represented by more than one probe.

### KLK5 inhibits EMTs

**Figure 4:**
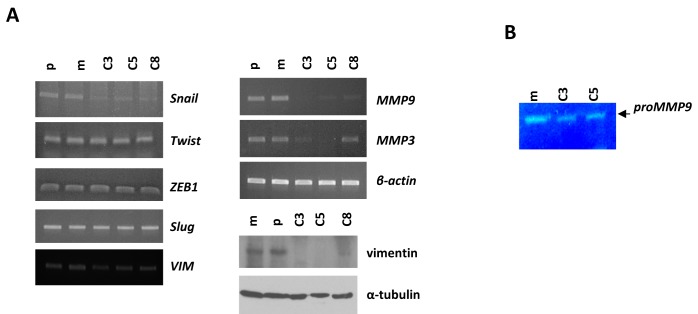
KLK5 expression is associated with suppression of EMTs A, Restoration of KLK5 expression represses mesenchymal markers and MMPs. Expression of SNAILl, TWIST, ZEB1, SLUG, MMP9, and MMP3 were analyzed by RT-PCR. VIM was measured by RT-PCR and western blot. B, Gelatin zymography showed that the proteolytic activity of secreted MMP-9 is inhibited by KLK5 (C3, C5).

It is well-established that the phenotypic changes associated with EMTs include increased motility, enhanced production of ECM-degrading enzymes, such as MMP-9 and MMP-3 and disruption of E-cadherin-mediated cell-cell adhesion. Based on our finding that KLK5 re-expression resulted in reduced motility and reduced active RhoA (see below), we asked whether the expression of EMT markers may be altered. We found that vimentin (*VIM*) was highly repressed in KLK5 transfectants at the protein and also the mRNA levels to a lesser extent (Fig.4A). Re-expression of E-cadherin could not be detected by RT-PCR (data not shown). Of the embryonic transcription factors SNAIL1, ZEB1, SLUG and TWIST, shown to induce EMTs, only *SNAIL1* was significantly downregulated (Fig.4A), while *ZEB1*, *SLUG* and *TWIST* were unchanged. It is known that SNAIL1 regulates the expression of MMP-9 [21], which in turn cooperates with SNAIL1 to induce EMTs [22]. By RT-PCR and gelatin zymography we found that both the expression and the proteolytic activity of MMP-9, respectively, were significantly reduced upon re-expression of KLK5 (Fig.4A and B). Furthermore, we found that the MMP-3/stromelysin-1, which is regulated by SNAIL1 [23], was also repressed (Fig.4A). These findings link KLK5 with the suppression of EMTs.

### Cholesterol homeostasis in KLK5-expressing cells

**Figure 5:**
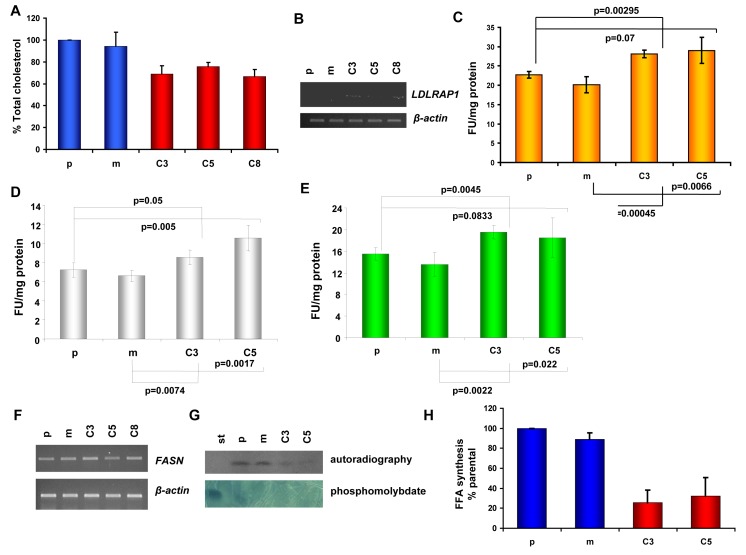
Cholesterol homeostasis in KLK5-expressing cells A, The total cholesterol content of KLK5-expressing MDA-MB-231 cells (C3, C5, and C8) is lower compared to mock and parental controls. B, LDLRAP1 is induced by KLK5 as shown by RT-PCR. C, DiI-LDL binding and internalization. D, DiI-LDL binding. E, internalization. Internalization or overall binding and internalization are increased in KLK5-expressing cells by approximately 25-30% compared to parental and mock controls indicating that cells are cholesterol-deprived and that the LDLR pathway is functional in these cells. F, Expression of fatty acid synthase (FASN) in KLK5 transfectants was not altered. G, Fatty acid synthesis rates were measured by administration of radiolabelled acetate / incorporation of [1-^14^C]-acetate and the products were analyzed by TLC and visualized by autoradiography (upper) and by phosphomolybdate staining (lower). One of three independent experiments is shown. H, Quantification of the free fatty acid (FFA) synthesis rates determined in G. Bars indicate SD. st, standard.

Given that KLK5 altered multiple genes with established roles in the mevalonate pathway and the expression profile was compatible with a state of cholesterol starvation, we focused on studying any functional consequences on cholesterol homeostasis. We show that total cellular cholesterol was lower by approximately 20-25% in KLK5-expressing MDA-MB-231 cells (Fig.5A). It is well-established that cholesterol deprivation induces the genes encoding the LDLR, PCSK9 and LDLRAP1 proteins which are involved in cholesterol uptake [24]. In accordance, both LDLR (1.97-fold) and PCSK9 (8.69-fold) were among the unregulated genes identified by microarray profiling (Table 1 and Sup Table S2), and induction of LDLRAP1 (2.6-fold), which is necessary for receptor endocytosis, was confirmed by semi-quantitative RT-PCR (Fig.5B). Following, we assessed the function of LDL by measuring DiI-LDL binding and endocytosis. As can be seen in Fig.5C-E both the binding of LDL to the surface of KLK5-expressing cells and endocytosis were found increased by 25%.

### Fatty acids synthesis

Because lipids are also synthesized *via* the mevalonate pathway, we tested whether fatty acid biosynthesis was altered. While the levels of FASN mRNA remained unaltered (Fig. 5F), KLK5-expressing cells exhibited significantly lower rates of free fatty acid (FFA) biosynthesis (Fig. 4G and H), which may be linked to suppressed malignancy since it is well-established that decreased FFA is associated with reduced aggressiveness of tumor cells [25].

### Sensitization to statins

**Figure 6:**
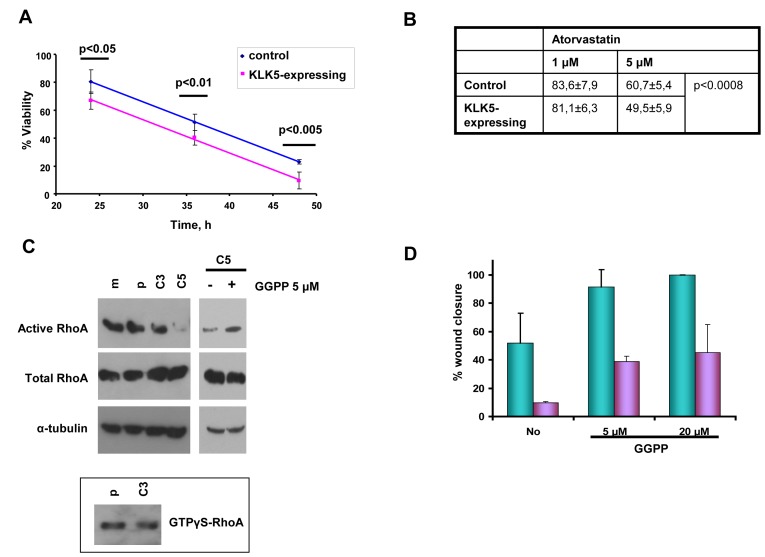
KLK5 may suppress malignancy by inhibiting RhoA signaling and EMTs Sensitization to statins. A, and B, KLK5 sensitizes MDA-MB-231 breast cancer cells to statins, i.e. 1 μM simvastatin (panel A) and atorvastatin (panel B). Cell cytotoxicity was measured by the MTT assay as described in Materials and Methods. Bars indicate SD from at least three independent experiments each conducted in quadruplicate. KLK5 reduces motility and anchorage-independent growth of MDA-MB-231 cells likely by inhibiting RhoA activation. KLK5 reduces the amount of active RhoA (C) and this suppression of active RhoA is reversed when C5 cells are treated with GGPP. Active RhoA was pulled-down on Rhotekin beads and detected by Western blotting. Total RhoA was analyzed to ensure equal loading. GTPγS-loaded RhoA was used as positive control. D, GGPP increases the motility of C5 cells in a dose-dependent manner either in the presence (24h, green) or absence (5h, blue) of serum.

We found that both simvastatin and atorvastatin were more cytotoxic to KLK5 transfectants than to parental and mock controls (Fig. 6A and 6B), indicating that lower cholesterol in *KLK5*-expressing cells sensitizes them to statins.

### KLK5 is associated with reduced active RhoA

We asked whether the observed suppression of the mevalonate pathway and, therefore, reduced synthesis of isoprenoids may affect the activity of signaling proteins that require isoprenylation for activation. We found that the levels of active RhoA transforming protein were diminished in KLK5-transfectants by more than 10-fold. As shown in Fig.6C (*upper*), significantly lower amounts of active RhoA (GTP-bound RhoA) were pulled-down from C3 compared with controls (parental, mock), while in C5 active RhoA was only barely detectable. Total RhoA was approximately the same in all cell populations (Fig.6C, *middle*). Addition of GGPP restored active RhoA (Fig.6C) but also increased the motility of C5 cells dose-dependently (Fig.6D) and anchorage-independent growth (data not shown). These results indicate that inhibited signaling *via* RhoA due to limited prenylation likely accounts for the observed suppression of malignancy in KLK5 transfectants.

**Figure 7:**
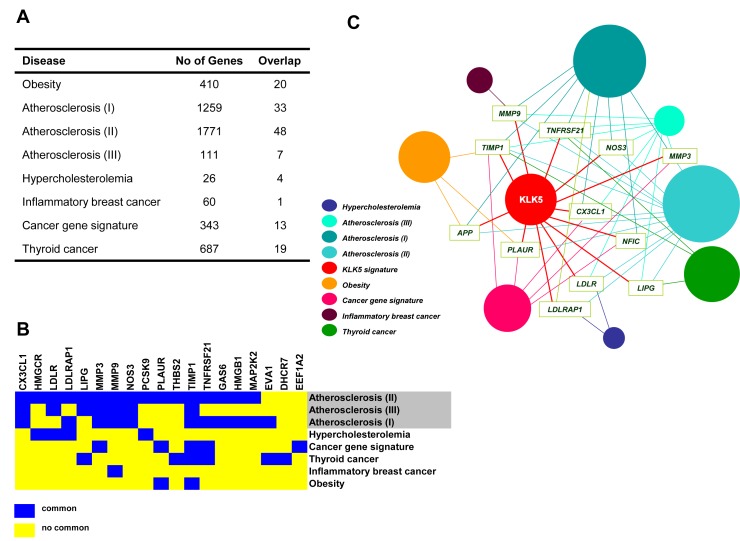
The “KLK5 signature” overlaps with gene signatures of cholesterol-associated diseases and cancer A, Disease gene lists established for obesity, atherosclerosis, hypercholesterolemia, and cancer were retrieved. In each case, the total number of genes is given in the middle column. The number of genes common in the “KLK5 signature” and in each given disease list are shown in the right column. The corresponding references are detailed in Materials and Methods. The complete “hypercholesterolemia gene list” was derived here and is provided in Sup Table S5. B, The heat map representation of 19 genes shared by the “KLK5 signature” and established signatures for atherosclerosis (II, III, I), hypercholesterolemia, cancers, and obesity shows marked overlap between the “KLK5 signature” and the “atherosclerosis signature”. The full list is provided in Sup Tables S5 and S6. C, Construction of a human disease network using the data given in Sup Tables S2, S5 and S6. Genes with the highest connectivity (≥4) relevant to more than four disorders were used as disease linkers and are indicated with the gene symbol. Each circle corresponds to a distinct disease signature (coloring is shown on bottom left) including the “KLK5 signature” identified here for breast cancer. The size of the diameter of each circle is proportional to the number of genes associated with a given disorder.

## DISCUSSION

We aimed to investigate whether re-expression of the KLK5 protease - reportedly inactivated in the vast majority of mammary carcinomas - may pose functional consequences for tumor growth and/or dissemination. For the first time, we present evidence that KLK5 inhibits breast cancer *in vitro* and *in vivo*, consistent with the emerging concept that certain proteases act as tumor suppressors [10]. Furthermore, we show that inhibition of EMTs may underlie the cancer suppressing effects of KLK5. Recently, it was reported that reactivation of KLK5 reverted the malignancy of ES-2 ovarian cancer cells [26]; however, no mechanistic insight has been provided. A novel and very intriguing finding of our study is that expression of KLK5 is associated with alteration in several genes encoding enzymes and regulatory proteins of the mevalonate pathway, including the induction of SREBF1 transcription factor. This translated into lower cell cholesterol and higher LDL uptake, indicating inhibition of the mevalonate pathway. Similar gene alterations are induced by statins, the widely prescribed cholesterol-lowering drugs, which also activate the SREBFs and their target genes/proteins engaged in cholesterol biosynthesis and uptake [6, 27].

It is currently well-established that altered metabolism is a hallmark of cancer cells compared to their normal counterparts [28-29], however, the intricacies of how metabolic pathways interconnect with oncogenic signaling remain largely unexplored. A central and well-described metabolic route is the mevalonate pathway that leads to the generation of isoprenoids required for cholesterol biosynthesis, post-translational modifications of proteins, synthesis of ubiquinone, etc. The rate-limiting step of this biosynthetic pathway is the reduction of 3-hydroxy-3-methyl-glutaryl-CoA catalyzed by the enzyme HMGCR [6], the pharmacological target of statins. Unexpectedly, results of epidemiological and clinical trials revealed that statins may prevent the development of different types of cancer [30]. Moreover, it was shown recently that dysregulation of the mevalonate pathway, achieved by ectopic expression of HMGCR, is causally linked to malignant transformation [7], thus pinpointing to HMGCR being a candidate metabolic oncogene. Very recently, increased cholesterol levels were detected in castrate-resistant prostate cancer, which were associated with downregulation of the TERE1 tumor suppressor [31]. Interestingly, inhibition of SREBP1 or its downstream target FASN sensitizes cancer cells to death ligands, a finding that may open new therapeutic approaches [32].

It is also well-known that statins inhibit Rho/Ras activation by impairing protein (iso)prenylation-farnesylation and geranylgeranylation, as isoprenoids are intermediate products of the mevalonate pathway (Fig.3A). This prompted the developement of geranyltransferase or farnesyltransferase inhibitors as potential anticancer drugs [33]. These inhibitors can act by altering oncogenic signaling. Also, they can attenuate the growth of tumors by modulating the immune response against tumor cells, as shown for the farnesyltransferase inhibitor salirasib, which suppressed glioblastoma growth in mice by increasing antitumor T-cell reactivity [34]. Consistent with inhibited biosynthesis of isoprenoids we detected markedly reduced levels of active (*i.e.* geranylgeranylated) RhoA in KLK5-expressing breast cancer cells. It should be mentioned that we did not detect changes in Ras activity (data not shown). Nonetheless, earlier studies showed that statin-induced anticancer effects on MDA-MB-231 are mainly mediated by RhoA [35-36] pointing to a dominant role of RhoA over Ras in determining the oncogenic potential of these cells [37]. Generally, it is well-established that Rho small GTPases coordinate many aspects of cell motility through reorganization of the actin cytoskeleton and changes in gene transcription. RhoA, in particular, upregulates the expression of MMP-9 in certain cells, including MDA-MB-231 cells, thus, enhancing their invasive potential [38]. RhoA is highly upregulated in breast tumors but barely detectable in normal adjacent tissues [39]. We found that re-expression of KLK5 not only reduces active RhoA but also the expression and proteolytic activity of secreted MMP-9, which both likely account for reduced malignancy. Furthermore, repression of RhoA activity could account for the observed inhibition of MMP-9, since it has been shown that cholesterol deprivation by simvastatin treatment reduces the expression and secretion of MMP-9 [40] *via* inhibition of RhoA signaling. It should be emphasized that suppression of oncogenic signaling by inhibition of protein prenylation prompted the development of farnesylation and geranylgeranylation inhibitors as potential anticancer drugs [41].

The mechanism(s) by which KLK5 infers the identified gene alterations merit further investigation. Intrigued by recent findings linking the high activity of the mevalonate pathway to enhanced transcription by mutant p53 in MDA-MB-231 [42] but potentially in breast cancer stem cells as well [43], we tested but found no significant changes in p53 mRNA or protein levels (Sup Fig. S3). It has been also reported that simvastatin-induced cholesterol deprivation upregulates p53 in MDA-MB-231 [44]. Although we were able to replicate this result, we did not observe the same effect with atorvastatin indicating that p53 induction may not be solely linked with cholesterol deprivation (Sup Fig. S3). Alternatively, KLK5 could induce endoplasmic reticulum stress that in turn activates *SREBF1*, *SREBF1*-responsive genes and *CASP4*, *TRIM13* [45-46] as found here. Current knowledge on the physiological function(s) of the KLK5 protease is restricted to its central role in the regulation of skin desquamation, while its hyperactivation has been causally linked to severe overdesquamating and inflammatory skin disorders [17, 47]. KLKs affect signaling by proteolytically activating the PARs, and KLK5 has been shown by *in vitro* proteolysis to activate PAR2 [11]. Further, KLK14 has been linked to oncogenic signaling in colon cancer where it activates PAR2 to induce ERK1/2 signaling and cellular proliferation [48]. The findings presented here provide novel insight into the emerging roles of KLKs in cell signaling *via* mechanisms not described previously. In conclusion, we show that KLK5 attenuates tumorigenicity of invasive breast cancer cells *in vitro* and *in vivo* and may represent a putative *Class II* tumor suppressor. The mechanisms underlying the cancer suppressing effects of KLK5 merit further investigation. We report for the first time, that intriguingly a secreted protease may suppress malignancy by downregulating the mevalonate pathway with important implications for pharmacological intervention.

## METHODS

### Materials

Synthetic oligonucleotides were obtained from VBC Biotech (Austria) or Invitrogen, anti-RhoA from Santa Cruz (Santa Cruz, CA) and anti-KLK5 from R&D Systems (Minneapolis, MN). All other antibodies were from Sigma (Saint Louis, MO). All other chemicals were of analytical grade and were obtained from Sigma or Merck (Darmstadt, Germany).

### Cell culture and stable transfections

The MDA-MB-231 cell line was obtained from the American Type Culture Collection. Cells were cultured as described [49]. The cDNA encoding preproKLK5 was amplified by PCR from a full-length *KLK5* cDNA using gene-specific primers (Sup Table S7) and cloned into the pcDNA3.1(+) vector (Invitrogen, Carlsbad CA). Plasmids were purified (Qiagen, Valencia, CA) and confirmed by DNA sequencing (ACGT, Toronto, Canada). Stably transfected MDA-MB-231 cells were selected with G418.

### *In vitro* cell assays

Growth curves, anchorage-independent growth and cellular motilities were studied as described [49]. In wound scratch assays, area quantification was performed using ImageJ (http://rsbweb.nih.gov/ij/).

### Tumor xenografts

2×10^6^ cells were resuspended in 100 μL of PBS and injected bilaterally into the mammary fat-pad of 6 week-old female SCID mice. Mice were examined on alternate days for the presence of palpable tumors. Tumors were allowed to grow for the indicated times and their sizes were measured double-blinded. Finally, mice were sacrificed, tumors were excised and photographed. Tumor volumes were calculated using the formula: ½ × height × width × length. All mouse experiments were conducted in duplicate and in compliance with the EU/Greek legislation and the approved guidelines of our institutions for animal handling.

### RT-PCR

Total RNA was reverse-transcribed into cDNA with Superscript RT II (Invitrogen) and amplified by PCR; primers and conditions are shown in Sup Table S7. Products were resolved stained with ethidium bromide and photographed.

### RhoA activation

Active RhoA was pulled-down from whole cell lysates by binding onto Rhotekin-GST beads (Cytoskeleton, Denver, CO). Cells were grown to 80-90% confluence, rinsed with ice-cold PBS and immediately lysed in the presence of protease inhibitors. Lysates (~2 mg) were clarified by centrifugation and then incubated with 25 μl beads (83 μg of protein) for 1 h at 4°C. Beads were collected and washed, then, proteins were eluted and detected by Western blot.

### Zymography

SFCM were collected at 70% confluence following incubation for 48 h and concentrated 8-fold with 10 kDa cutoff spin filters (Amicon). Samples containing equal amounts of total protein were resolved on 12% SDS-PAGE containing 0.1% gelatin under non-reducing conditions. To remove SDS, gels were washed twice with 50 mM Tris.HCl, pH 7.5, 5 mM CaCl_2_, 2.5% Triton X-100 and finally without Triton X-100. For gelatin cleavage, gels were incubated (at 37°C for 20 h) in the same solution containing 0.1% Triton X-100 and then stained with Amidoblack.

### Western blotting

Cells were lysed in RIPA (PBS containing 1% NP-40, 0.5% sodium deoxycholate, 0.1% SDS). 100 μg of total protein were resolved on 12% SDS-PAGE, and electroblotted onto PVDF membrane (Millipore, Billerica, MA). Monoclonal antibodies (anti-vimentin, anti-α-tubulin, anti-RhoA, anti-SREBP1) were added at 1:2,000 dilution and secondary antibodies at 1:3,000. Immunoreactive bands were detected with West Pico ECL (Pierce, Rockford IL). For KLK5 detection, proteins contained in 20 ml of SFCM were precipitated with phenol [50], dissolved in loading dye and analyzed. The anti-KLK5 antibody was used at a dilution of 1:1,000.

### Cytotoxicity

Activation of simvastatin (an inactive lactone prodrug) *via* basic hydrolysis was carried out as following: 25 mg of simvastatin (Sigma) were dissolved in 0.5 ml ethanol and 0.407 ml 1 M NaOH; the reaction was allowed to take place for 30 min at room temperature. HCl was added to pH 7.0 and the stock solution of 10 mM in PBS was stored at −20°C. For cytotoxicity assays, 10^5^xcells were allowed to adhere and grow on 24-well plates for 24 h. Activated simvastatin or atorvastatin (Sigma) was added and cells were incubated for the required times. Subsequently, the medium was removed, cells were washed with PBS and fresh medium containing 0.5 mg/ml MTT was added. Cells were further incubated for 1 h, medium was removed and the insoluble formazan crystals were dissolved in 100% DMSO for 30 min at 37°C. Absorbance was recorded at 570 nm. Cytotoxicity was determined using the formula: (A570_treated cells_-A570_background_)/ (A570_untreated cells_-A570_background_)*100%. Background absorbance was measured in the absence of cells.

### Microarray profiling and data analysis

For microarray analysis, MDA-MB-231 parental, pcDNA3.1(+) vector-transfected (mock) and pcDNA3.1(+)/preproKLK5-transfected cells were grown to 70% confluence, harvested in PBS and total RNA was extracted with Qiagen RNeasy. The integrity of RNA was confirmed by electrophoresis and its concentration and purity were determined spectrophotometrically. The synthesis of cDNA and biotinylated cRNA was performed using Illumina TotalPrep RNA Amplification (Illumina, San Diego, California) and 500 ng total RNA. Hybridization onto Illumina Human WG-6 V3 BeadChips was carried out according to manufacturer's instructions. Data were pre-processed and normalized with the lumi algorithm implemented on FlexArray software (http://genomequebec.mcgill.ca/FlexArray). Log_2_-based transformation was applied for variance stabilization and normalization was performed using the Quantile method. Genes with detection score higher than 0.99 were removed. To derive the differentially expressed genes, statistical analysis compared pooled KLK5-expressing cells (clones C3 and C5) and KLK5-negative controls (parental, mock) with the ARMADA software [51] using t-test with a p-value cutoff set at 0.05. Genes with a fold-change value below |0.5| (in log_2_ scale) were removed. To identify groups of genes referring to the same biological process or cellular biochemical pathway, the list of differentially expressed genes was analyzed according to Gene Ontology Terms (GOTs) using StRAnGER software [52].

### Patient data analysis

Microarray data identified by Oncomine [53] were manually extracted and introduced into Microcal Origin 8 or Microsoft Excel for generation of graphs and for statistical analysis.

### Identification of common nodal genes

Disease gene sets were retrieved from published studies. These included the “343 gene set” of the cancer gene signature [19], the “60 gene set” of inflammatory breast cancer [54], three different gene sets of atherosclerosis: the “1,259 gene set” (experimental), the “1,771 gene set” (literature) and the “111 gene set” (established) [55], the “410 gene set” of obesity [56], and the “687 gene set” of thyroid cancer [57]. For hypercholesterolemia, we generated a new list by thorough text mining which included only those genes that are directly related to the disease and do not represent changes that arise as secondary effects of high cholesterol.

### Interaction networks and transcription factor prediction

Microarray data were introduced into IPA (http://www.ingenuity.com/) to generate networks each of which contained 35 molecules and for transcription factor predictions.

### Cholesterol content

Total cellular cholesterol was determined based on the reaction of cholesterol or cholesteryl esters with Fe^3+^ in H_2_SO_4_. Briefly, cells were washed and harvested. Cholesterol was extracted with CHCl_3_:CH_3_OH 2:1 for 1 h, then, the organic layer was evaporated to dryness at 55°C, 1.5 ml of FeCl_3_, CH_3_COOH reagent was added to the pellet for 10 min, followed by 1 ml of H_2_SO_4_ for 45 min at 27°C in the dark [58]. Total cholesterol was determined from the absorbance at 560 nm using standards and was normalized against protein. Each experiment was performed in triplicate.

### LDL uptake

The assay measures the uptake of DiI-LDL, a fluorescent derivative of LDL, and was carried out as described [59]. Briefly, cells were grown in 24-wells and DiI-LDL was added to 5 μg/ml in SFCM for 2 h at 37°C (binding and uptake) or 4°C (binding only). Cells were washed with PBS and lysed in 0.1 M NaOH containing 0.1 % SDS. The difference between the fluorescence at 37°C and 4°C is an indicator of the internalization effect only. Fluorescence was measured at 612 nm (emission)/544 nm (excitation) and normalized against protein. Each experiment was carried out in triplicate.

### Fatty acid synthesis

The assay is based on the incorporation of the [1-^14^C]-acetate precursor during lipid biosynthesis [59]. Cells were grown in 100 mm plates and sodium [1-^14^C]-acetate was added at 1 μCi/ml in SFCM for 3 h at 37°C. Then, the medium was removed, cells were harvested in 250 μl RIPA and lipids were extracted in 1 ml CHCl_3_:CH_3_OH 2:1. The organic layer was allowed to evaporate, lipids were re-dissolved in 20 μl CHCl_3_:CH_3_OH 2:1 and resolved by TLC with developing solvent petroleum ether:diethylether:acetic acid (80:30:1). Standards were co-chromatographed. Incorporation of the [1-^14^C]-acetate into fatty acids was quantified by autoradiography and visualized with phosphomolybdic acid.

## SUPPLEMENTARY FIGURES AND TABLES





## References

[1] Brattsand M, Egelrud T (1999). Purification, molecular cloning, and expression of a human stratum corneum trypsin-like serine protease with possible function in desquamation. J Biol Chem.

[2] Michael IP, Sotiropoulou G, Pampalakis G, Magklara A, Ghosh M, Wasney G, Diamandis EP (2005). Biochemical and enzymatic characterization of human kallikrein 5 (hK5), a novel serine protease potentially involved in cancer progression. J Biol Chem.

[3] Yousef GM, Yacoub GM, Polymeris ME, Popalis C, Soosaipillai A, Diamandis EP (2004). Kallikrein gene downregulation in breast cancer. Br J Cancer.

[4] Li X, Liu J, Wang Y, Zhang L, Ning L, Feng Y (2009). Parallel underexpression of kallikrein 5 and kallikrein 7 mRNA in breast malignancies. Cancer Sci.

[5] Feng Y, Li X, Sun B, Wang Y, Zhang L, Pan X, Chen X, Wang X, Wang J, Hao X (2010). Evidence for a transcriptional signature of breast cancer. Breast Cancer Res Treat.

[6] Goldstein JL, Brown MS (1990). Regulation of the mevalonate pathway. Nature.

[7] Clendening JW, Pandyra A, Boutros PC, El Ghamrasni S, Khosravi F, Trentin GA, Martirosyan A, Hakem A, Hakem R, Jurisica I, Penn LZ (2010). Dysregulation of the mevalonate pathway promotes transformation. Proc Natl Acad Sci USA.

[8] Gorin A, Gabitova L, Astsaturov I (2012). Regulation of cholesterol biosynthesis and cancer singaling. Curr Opin Pharmacol.

[9] López-Otín C, Hunter T (2010). The regulatory crosstalk between kinases and proteases in cancer. Nat Rev Cancer.

[10] López-Otín C, Matrisian LM (2007). Emerging roles of proteases in tumour suppression. Nat Rev Cancer.

[11] Oikonomopoulou K, Hansen KK, Saifeddine M, Vergnolle N, Tea I, Blaber M, Blaber SI, Scarisbrick I, Diamandis EP, Hollenberg MD (2006). Kallikrein-mediated cell signaling: targeting proteinase-activated receptors (PARs). Biol Chem.

[12] Ramachandran R, Noorbakhsh F, Defea K, Hollenberg MD (2012). Targeting proteinase-activated receptors: therapeutic potential and challenges. Nat Rev Drug Discov.

[13] Perou CM, Sørlie T, Eisen MB, van de Rijn M, Jeffrey SS, Rees CA, Pollack JR, Ross DT, Johnsen H, Akslen LA, Fluge O, Pergamenschikov A (2000). Molecular portraits of human breast tumours. Nature.

[14] Sørlie T, Perou C, Tibshirani R, Aas T, Geisler S, Johnsen H, Hastie T, Eisen MB, van de Rijn M, Jeffrey SS, Thorsen T, Quist H (2001). Gene expression patterns of breast carcinomas distinguish tumor subclasses with clinical implications. Proc Natl Acad Sci USA.

[15] Finak G, Bertos N, Pepin F, Sadekova S, Souleimanova M, Zhao H, Chen H, Omeroglu G, Meterissian S, Omeroglu A, Hallett M, Park M (2008). Stromal gene expression predicts clinical outcome in breast cancer. Nat Med.

[16] Grigoriadis A, Mackay A, Reis-Filho JS, Steele D, Iseli C, Stevenson BJ, Jongeneel CV, Valgeirsson H, Fenwick K, Iravani M, Leao M, Simpson AJ (2006). Establishment of the epithelial-specific transcriptome of normal and malignant human breast cells based on MPSS and array expression data. Breast Cancer Res.

[17] Sales KU, Masedunskas A, Bey AL, Rasmussen AL, Weigert R, List K, Szabo R, Overbeek PA, Bugge TH (2010). Matriptase initiates activation of epidermal pro-kalikrein and disease onset in a mouse model of Netherton syndrome. Nat Genet.

[18] Amemiya-Kudo M, Shimano H, Yoshikawa T, Yahagi N, Hasty AH, Okazaki H, Tamura Y, Shionoiri F, Iizuka Y, Ohashi K, Osuga J, Harada K (2000). Promoter analysis of the mouse sterol regulatory element-binding protein-1c gene. J Biol Chem.

[19] Hirsch HA, Iliopoulos D, Joshi A, Zhang Y, Jaeger SA, Bulyk M, Tsichlis PN, Shirley Liu X, Struhl K (2010). A transcriptional signature and common gene networks link cancer and lipid metabolism and diverse human diseases. Cancer Cell.

[20] Goldstein JL, DeBose-Boyd RA, Brown MS (2006). Protein sensors for membrane sterols. Cell.

[21] Jordá M, Olmeda D, Vinyals A, Valero E, Cubillo E, Llorens A, Cano A, Fabra A (2005). Upregulation of MMP-9 in MDCK epithelial cell line in response to expression of the Snail transcription factor. J Cell Sci.

[22] Lin CY, Tsai PH, Kandaswami CC, Lee PP, Huang CJ, Hwang JJ, Lee MT (2011). Matrix metalloproteinase-9 cooperates with transcription factor Snail to induce epithelial-mesenchymal transition. Cancer Sci.

[23] Haraguchi M, Okubo T, Miyashita Y, Miyamoto Y, Hayashi M, Crotti T, McHugh KP, Ozawa M (2008). Snail regulates cell-matrix adhesion by regulation of the expression of integrins and basement membrane proteins. J Biol Chem.

[24] Soutar AK, Naoumova RP (2007). Mechanisms of disease: genetic causes of familial hypercholesterolemia. Nat Clin Pract Cardiovasc Med.

[25] Menendez JA, Lupu R (2007). Fatty acid synthase and the lipogenic phenotype in cancer pathogenesis. Nat Rev Cancer.

[26] Pépin D, Shao ZQ, Huppé G, Wakefield A, Chu CW, Sharif Z, Vanderhyden BC (2011). Kallkreins 5, 6 and 10 differentially alter pathophysiology and overall survival in an ovarian cancer xenograft model. PLoS One.

[27] Dubuc G, Chamberland A, Wassef H, Davignon J, Seidah NG, Bernier L, Prat A (2004) Statins upregulate PCSK9, the gene encoding the proprotein convertase neural apoptosis-regulated convertase-1 implicated in familial hypercholesterolemia. Arterioscler Thromb Vasc Biol.

[28] Hanahan D, Weinberg RA (2011) Hallmarks of cancer: the next generation. Cell.

[29] Cairns RA, Harris IS, Mak TW (2011). Regulation of cancer cell metabolism. Nat Rev Cancer.

[30] Demierre MF, Higgins PDR, Gruber SB, Hawk E, Lippman SM (2005). Statins and cancer prevention. Nat Rev Cancer.

[31] Fredericks WJ, Sepulveda J, Lai P, Tomaszewski JE, Lin MF, McGarvey T, Rauscher FJ, Malkowicz SB (2013). The tumor suppressor TERE1 (UBIAD1) prenyltransferase regulates the elevated cholesterol phenotype in castration resistant prostate cancer by controlling a program of ligand dependent SXR target genes. Oncotarget.

[32] Eberhard Y, Gronda M, Hurren R, Datti A, MacLean N, Ketela T, Moffat J, Wrana JL, Schimmer AD (2011). Inhibition of SREBP1 sensitizes cells to death ligands. Oncotarget.

[33] Berndt N, Hamilton AD, Sebti M (2011). Targeting protein prenylation for cancer therapy. Nat Rev Cancer.

[34] Aizman E, Mor A, Levy A, George J, Kloog Y (2012). Ras inhibition by FTS attenuates brain tumor growth in mice by direct antitumor activity and enhanced reactivity of cytotoxic lymphocytes. Oncotarget.

[35] Klawitter J, Shokati T, Moll V, Christians U, Klawitter J (2010). Effects of lovastain on breast cancer cells: a proteo-metabonomic study. Breast Cancer Res.

[36] Denoyelle C, Vasse M, Körner M, Mishal Z, Ganné F, Vanier JP, Soria J, Soria C (2001). Cerivastatin, an inhibitor of HMG-CoA reductase, inhibits the signaling pathways involved in the invasiveness and metastatic properties of highly invasive breast cancer cell lines: an in vitro study. Carcinogenesis.

[37] Denoyelle C, Albanese P, Uzan G, Hong L, Vannier JP, Soria J, Soria C (2003). Molecular mechanism of the anti-cancer activity of cerivastatin, and inhibitor of HMG-CaA reductase, on aggressive human breast cancer cells. Cell Signal.

[38] Pillé JY, Denoyelle C, Varet J, Bertrand JR, Soria J, Opolon J, Lu H, Pritchard LL, Vannier JP, Malvy C, Soria C, Li H (2005). Anti-RhoA and anti-RhoC siRNAs inhibit the proliferation and invasiveness of MDA-MB-231 breast cancer cells in vitro and in vivo. Mol Ther.

[39] Fritz G, Just I, Kaina B (1999). Rho GTPases are over-expressed in human tumors. Int J Cancer.

[40] Turner NA, O'Regan DJ, Ball SG, Porter KE (2005). Simvastatin inhibits MMP-9 secretion from human saphenous vein smooth muscle cells by inhibiting the RhoA/ROCK pathway and reducing MMP-9 mRNA levels. FASEB J.

[41] Berndt N, Hamilton AD, Sebti SM (2011) Targeting protein prenylation for cancer therapy. Nat Rev Cancer.

[42] Freed-Pastor WA, Mizuno H, Zhao X, Langerød A, Moon SH, Rodriguez-Barrueco R, Barsotti A, Chicas A, Li W, Polotskaia A, Bissell MJ, Osborne TF, Tian B, Lowe SW, Silva JM, Børresen-Dale AL, Levine AJ, Bargonetti J, Prives C (2012). Mutant p53 disrupts mammary tissue architecture via the mevalonate pathway. Cell.

[43] Ginestier C, Charafe-Jauffret E, Birnbaum D (2012). p53 and cancer stem cells: the mevalonate connexion. Cell Cycle.

[44] Mandal CC, Ghosh-Choudhury N, Yoneda T, Choudhury GG, Ghosh-Choudhury N (2011). Simvastatin prevents skeletal metastasis of breast cancer by an antagonistic interplay between p53 and CD44. J Biol Chem.

[45] Tomar D, Singh R, Singh AK, Pandya CD, Singh R (2012). TRIM13 regulates ER stress induced autophage and clonogenic ability of the cells. Biochim Biophys Acta.

[46] Ghavami S, Yeganeh B, Stelmack GL, Kashani HH, Sharma P, Cunnington R, Rattan S, Bathe K, Klonisch T, Dixon IM, Freed DH, Halayko AJ (2012). Apoptosis, autophagy and ER stress in mevalonate cascade inhibition-induced cell death of human atrial fibroblasts. Cell Death Dis.

[47] Descargues P, Deraison C, Bonnart C, Kreft M, Kishibe M, Ishida-Yamamoto A, Elias P, Barrandon Y, Zambruno G, Sonnenberg A, Hovnanian A (2005). Spink5-deficient mice mimic Netherton syndrome through degradation of desmoglein 1 by epidermal protease hyperactivity. Nat Genet.

[48] Gratio V, Loriot C, Virca GD, Oikonomopoulou K, Walker F, Diamandis EP, Hollenberg MD, Darmoul D (2011) Kallikrein-related peptidase 14 acts on proteinase-activated receptor 2 to induce signaling pathway in colon cancer cells. Am J Pathol.

[49] Pampalakis G, Prosnikli E, Agalioti T, Vlahou A, Zoumpourlis V, Sotiropoulou G (2009). A tumor protective role for human kallikrein-related peptidase 6 in breast cancer mediated by inhibition of epithelial-to-mesenchymal transition. Cancer Res.

[50] Sauvé DM, Ho DT, Roberge M (1995). Concentration of dilute protein for gel electrophoresis. Anal Biochem.

[51] Chatziioannou A, Moulos P, Kolisis FN (2009). Gene ARMADA: an integrated multi-analysis platform for microarray data implemented in MATLAB. BMC Bioinformatics.

[52] Chatziioannou A, Moulos P (2011). Exploiting Statistical Methodologies and Controlled Vocabularies for Prioritized Functional Analysis of Genomic Experiments: the StRAnGER Web Application. Front Neurosci.

[53] Rhodes DR, Yu J, Shanker K, Deshpande N, Varambally R, Ghosh D, Barrette T, Pandey A, Chinnaiyan AM (2004). Oncomine: a cancer microarray database and integrated data-mining platform. Neoplasia.

[54] Lerebours F, Vacher S, Andrieu C, Espie M, Marty M, Lidereau R, Bieche I (2008). NF-kappa B genes have a major role in inflammatory breast cancer. BMC Cancer.

[55] Skogsberg J, Lundstrom J, Kovacs A, Nilsson R, Noori P, Maleki S, Köhler M, Hamsten A, Tegnér J, Björkegren J (2008). Transcriptional profiling uncovers a network of cholesterol-responsive atherosclerosis target genes. PLoS Genet.

[56] Lee YH, Nair S, Rousseau E, Allison DB, Page GP, Tataranni PA, Bogardus C, Permana PA (2005). Microarray profiling of isolated abdominal subcutaneous adipocytes from obese vs non-obese Pima Indians: increased expression of inflammation-related genes. Diabetologia.

[57] Delys L, Detours V, Franc B, Thomas G, Bogdanova T, Tronko M, Libert F, Dumont JE, Maenhautet C (2007). Gene expression and the biological phenotype of papillary thyroid carcinomas. Oncogene.

[58] Badzio T (1965). The possibilities of errors in determination of cholesterol in blood with FeCl3-reagent. Clin Chim Acta.

[59] Krycer JR, Kristiana I, Brown AJ (2009). Cholesterol homeostasis in two commonly used human prostate cancer cell-lines, LNCaP and PC-3. PLoS ONE.

