# Supplementary material for: The KLK5 protease suppresses breast cancer by repressing the mevalonate pathway
Source: Oncotarget. 2013 Sep 3;5(9):2390–403. doi: 10.18632/oncotarget.1235 (PMC4058013; doi:10.18632/oncotarget.1235)
Supplement: Supplementary file 1 [file oncotarget-05-2390-s001.pdf]

The KLK5 protease suppresses breast cancer by repressing the mevalonate pathway – Pampalakis et al

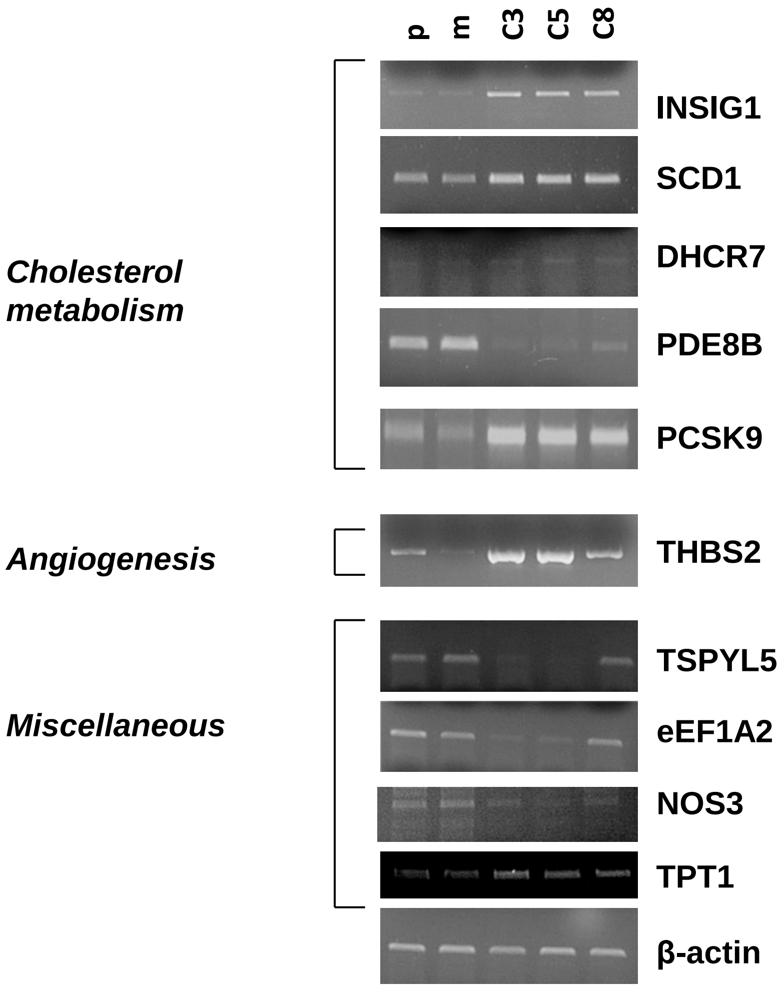

Supplementary Figure S1: Verification of the differential expression of selected genes identified in microarray profiling (Sup Table S2) by RT-PCR.

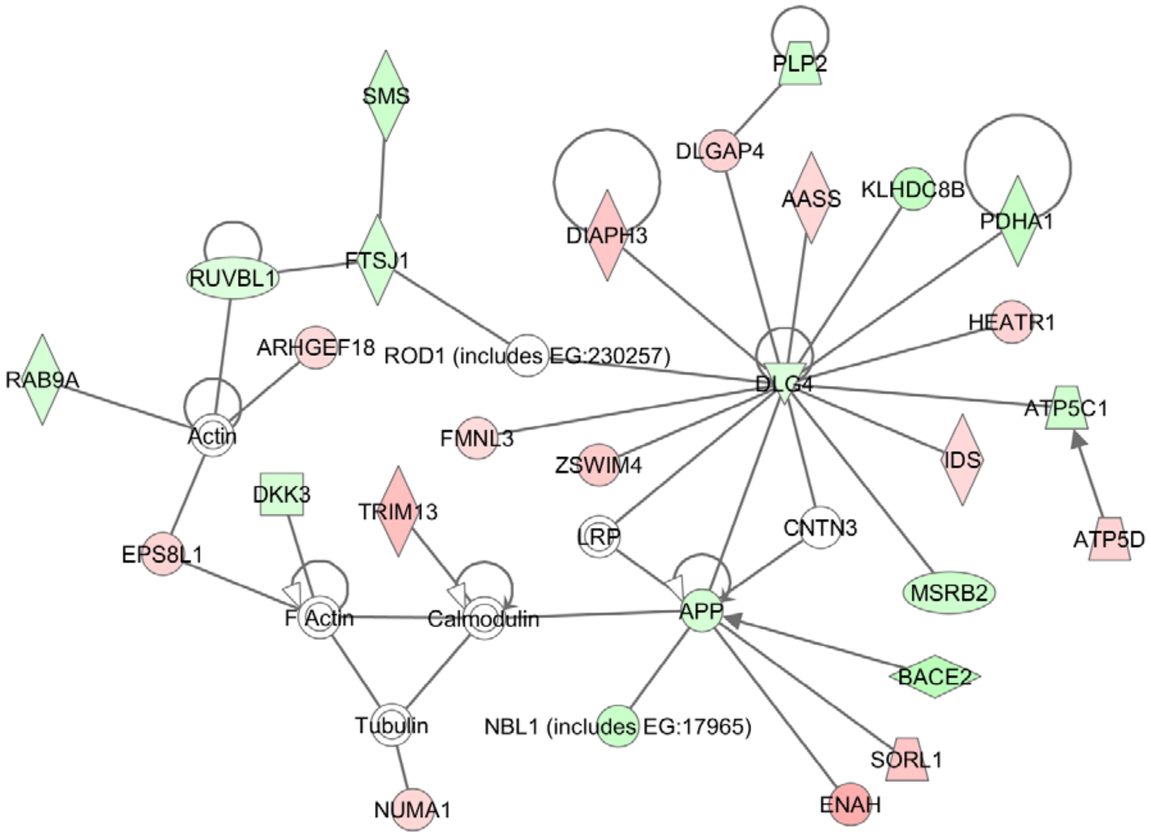

Supplementary Figure S2: Lipid metabolism network as it was generated by IPA.

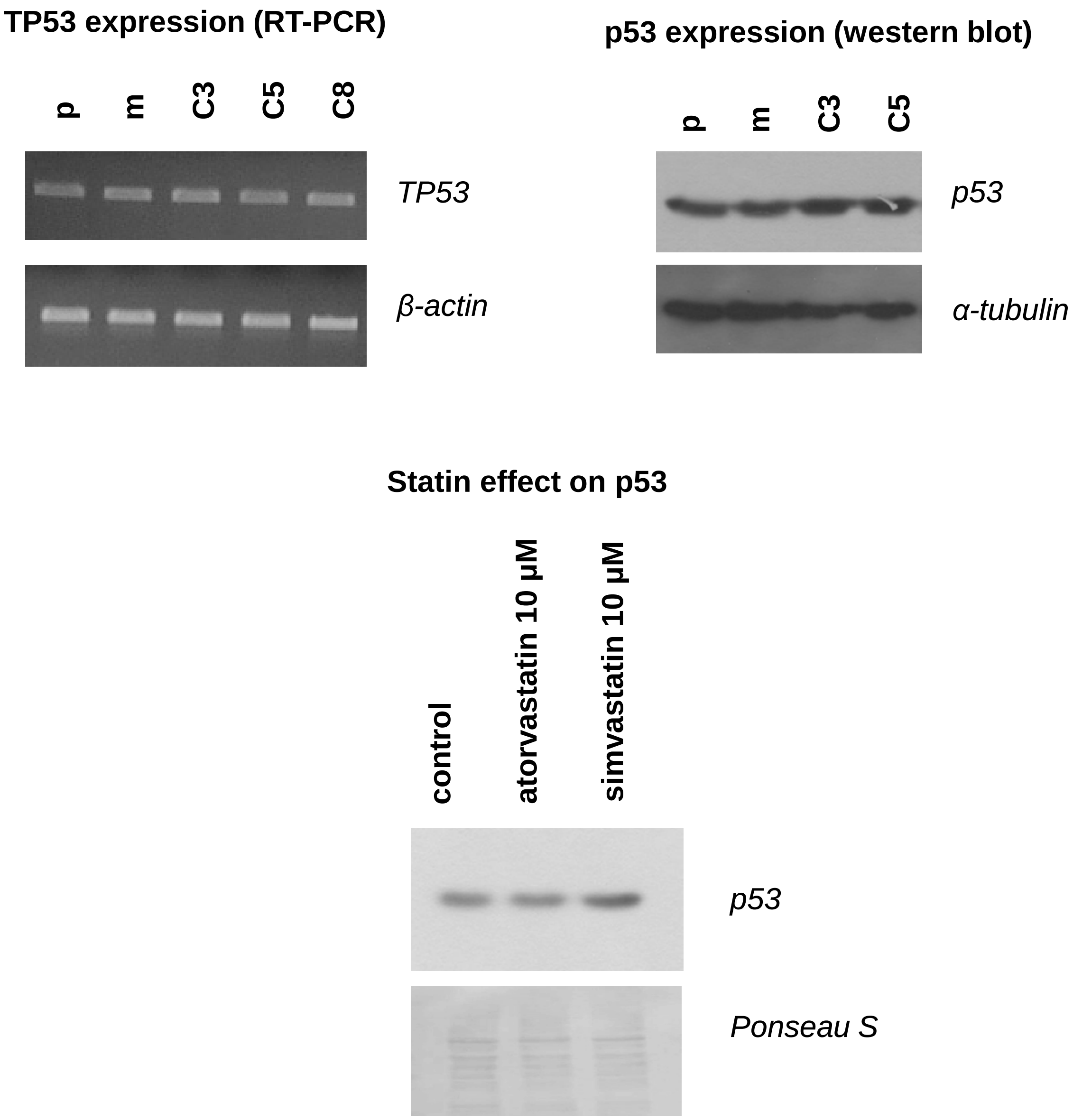

Supplementary Figure S3: Expression of TP53 and p53. The expression TP53 and p53 was measured by RT-PCR and western blot respectively (upper left and right). No significant alterations were found. Simvastatin was able to induce the expression of TP53 in MDA-MB-231 while atorvastatin was not (lower).
